# Beyond the numbers: Reimagining procedural proficiency in emergency medicine residencies

**DOI:** 10.1002/aet2.10920

**Published:** 2023-11-29

**Authors:** Michelle I. Suh, Carl Preiksaitis, Esther Chen

**Affiliations:** ^1^ University of Chicago Chicago Illinois USA; ^2^ Stanford University Palo Alto California USA; ^3^ University of California San Francisco/San Francisco General Hospital San Francisco California USA

“So, how many of these have you done before?” As supervising attendings, we often ask this question to the resident setting up for a procedure as we decide whether to grab our own set of sterile gloves. Yet, this question assumes that the number of times someone has performed a procedure reflects their comfort, knowledge, and skill in performing that procedure. As academic physicians tasked with training competent emergency physicians, we need to set a higher bar for procedural skill attainment. While case numbers may help with skill attainment, procedural practice with performance feedback and assessment are critical to helping the novice achieve procedural proficiency and becoming an expert.^1^ Already adopted by surgical specialties, proficiency‐based skills training represents a fundamental shift from quantity to quality. This requires establishing a proficiency criterion, providing objective and timely feedback to a trainee, and assessing a trainee's readiness for independent practice.^2^ Patient safety depends on our ability as emergency physicians to be procedurally proficient to provide skilled, competent care.

## THE CURRENT LANDSCAPE OF PROCEDURAL TRAINING

Procedural experience during emergency medicine (EM) training has primarily focused on procedural numbers across program types or trends in numbers over time.^3^, ^4^ During residency, trainees’ procedural skills are assessed biannually (Patient Care 8 of the EM Milestones) and case volume is measured as mandated by the EM Residency Review Committee.^5^, ^6^ A critical part of the EM resident portfolio, procedural logs satisfy the minimum procedural counts required by the EM Residency Review Committee.^6^ In this issue, Turner et al.^7^ questioned the adequacy of a minimum standard and highlighted the variability in trainee attempts needed to achieve proficiency in simulated cricothyrotomy, as measured by time to successful tracheal tube placement. Even though programs are required to measure their residents’ procedural milestones biannually,^3^ the approach by Turner et al. to procedural assessment is probably more than what most EM programs do in procedural assessment. However, is this sufficient to ensure procedural proficiency?

Developing proficiency in a procedure requires two things: (1) nontechnical skills, such as knowledge of procedural indications and complications, and (2) technical skills, often referred to as microsteps. However, we cannot know what we do not measure, and case logs only measure the number of cases done. Logging a pericardiocentesis performed in simulation lab does not necessarily mean that the resident is able to understand the indications for the procedure, how to set up for the procedure, or even if they can perform the procedure successfully a year later. Exposure as a proxy for learning dates back to Osler's *natural method of teaching*, codified in the procedural space by Halsted's famous “see one, do one, teach one.”^8^ However, there is increasing evidence to support simulation‐based medical education with deliberate practice as a more effective way of learning procedures.^9^ We—as physicians, training programs, governing bodies, and education researchers—owe it to our patients to embrace a more comprehensive approach to procedural proficiency.

## THE PATH FORWARD: EMPHASIZING QUALITY OVER QUANTITY

Governing bodies providing oversight over EM residency training could regularly reevaluate their standards for procedural competency beyond setting minimum requirements and general procedural milestone achievements. National professional EM organizations could help coordinate residency leaders to establish a proficiency criterion for each procedure and checklists with microskills that become a shared resource for both new and established programs.

Residency programs could support their learners in building and assessing their own procedural competency toward a proficiency criterion. Deliberate practice could be used to guide resident performance with clear, measurable objectives and focused feedback, and opportunities to practice in a safe environment, such as using simulation with task trainers.^10^ Assessment must accompany procedural teaching and practice for the resident to achieve proficiency. Mastery learning checklists with clear expectations can serve as objective assessment of resident procedural proficiency.^11^ We encourage programs to develop a longitudinal, comprehensive way to track resident progress, identify gaps, and confirm readiness for independent practice, particularly for high‐acuity low‐occurrence procedures.

## RISING ABOVE THE FLOOR: CULTIVATING LIFELONG LEARNERS

However, the onus should not just be on training programs. We agree with the call by Santen et al.^12^ that advocacy for lifelong learning should start in residency and continue beyond graduation. Residents themselves may start the process of being lifelong procedural learners, by setting personal goals, practicing technical skills, reflecting on knowledge gaps, and asking for feedback from trusted mentors. Mastery learning checklists may be used as guides with goalposts for independent study. Echoing Santen et al. in their commentary,^12^ we owe it to our patients to carry these skills past graduation, when we may no longer be required to demonstrate our competencies. We should be equipping learners with ways to ensure they continue to maintain their procedural skills.

Finally, and no less importantly, we issue a call to our collective community of education researchers and program leaders to innovate and investigate different ways to teach and assess procedural competency. What are the best practices for teaching procedures? How can we better assess procedural competency for specific procedures? How do we maintain retention of procedural skills? We can and should expect more from our learners than the floor set by minimum procedural numbers. Let's do better for our patients and help our EM trainees reach for the ceiling.

## CONFLICT OF INTEREST STATEMENT

The authors declare no conflicts of interest.
